# Application, performance and limitations of artificial intelligence for the diagnosis and prediction of oro-facial pain: a systematic review

**DOI:** 10.3389/froh.2026.1895438

**Published:** 2026-08-05

**Authors:** Sanjeev B. Khanagar, Areej Alfaifi, Oinam Gokulchandra Singh, Afnan Alayyash, Fayaz Ul Haq, Barrak Alsomaie, Sudarshan Bhat, Vineet Khinda, Satish Vishwanathaiah, Kiran Iyer

**Affiliations:** 1 Preventive Dental Science Department, College of Dentistry, King Saud bin Abdulaziz University for Health Sciences, Riyadh, Saudi Arabia; 2 King Abdullah International Medical Research Centre, Riyadh, Saudi Arabia; 3 Ministry of the National Guard Health Affairs, Riyadh, Saudi Arabia; 4 Department of Restorative and Prosthetic Dental Sciences, College of Dentistry, King Saud bin Abdulaziz University for Health Sciences, Riyadh, Saudi Arabia; 5 Radiological Sciences Program, College of Applied Medical Sciences, King Saud bin Abdulaziz University for Health Sciences, Riyadh, Saudi Arabia; 6 Department of Preventive Dentistry, College of Dentistry, Taif University, Taif, Saudi Arabia; 7 Department of Oral Aand Maxillofacial Surgery, Armed Forces Medical College, Pune, Maharashtra, India; 8 Department of Preventive Dental Sciences, Division of Pedodontics, College of Dentistry, Jazan University, Jazan, Saudi Arabia

**Keywords:** artificial intelligence, deep learning, diagnostic accuracy, machine learning, neural networks, orofacial pain, pain prediction, temporomandibular disorders

## Abstract

**Background:**

Oro-Facial Pain (OFP) disorders are a group of diseases characterized by overlapping clinical features leading to diagnostic and therapeutic challenges. Recent advances in artificial intelligence (AI) such as machine learning (ML) and deep learning (DL) have shown potential to improve the accuracy of diagnosis, prediction of pain and decision making in the clinic. The objective of this systematic review is to assess the use, diagnostic accuracy, and clinical utility of AI models in the diagnosis and prediction of OFP conditions.

**Methods:**

This systematic review was performed following the PRISMA-DTA guidelines and registered at PROSPERO (CRD420261322606). A comprehensive electronic search was conducted for studies published from January 1, 2000 to February 1, 2026 in PubMed, Scopus, Embase, Cochrane Library, Web of Science and Google Scholar. Eligible studies examined AI-based methods for the diagnosis, classification, localization or prediction of OFP conditions. Independent reviewers performed study selection, data extraction, and quality assessment. Methodological quality was assessed using the QUADAS-2 tool and the certainty of the evidence was assessed using GRADE approach. Qualitative synthesis was performed due to substantial heterogeneity in study design, datasets, AI architectures and outcome measures.

**Results:**

Twenty studies were included. These studies assessed AI applications for general diagnosis of orofacial pain, temporomandibular disorders, trigeminal neuralgia and facial pain syndromes, prediction of postoperative odontogenic pain, and localization of dental pain. The AI models were trained on heterogeneous data modalities including clinical records, questionnaires, thermography, radiographic imaging, MRI, neuroimaging and electronic health records. ML algorithms and artificial neural network (ANN) models demonstrated diagnostic accuracies ranging from 75% to 99%, demonstrating a potential for better performance on imaging-based and structured clinical datasets. Thermography-based ML models achieved the highest reported accuracy (99%) over other modalities, and MRI-based predictive models for temporomandibular disorders exhibited high discriminatory ability (AUC up to 0.899). Most studies were classified as low risk of bias in the patient selection and index test domains, but there were major concerns with regard to applicability and limited external validation in the reference standard domain. Overall certainty of evidence was rated as moderate.

**Conclusion:**

AI-based systems have great potential as adjunctive tools for the diagnosis, classification and prediction of OFP conditions, especially when using structured clinical and imaging datasets. However, the current evidence is limited by methodological heterogeneity, retrospective study design, and lack of external validation. Future research should focus on prospective multi-center studies, standardized datasets, explainable AI frameworks, and the integration of multimodal AI approaches into clinically applicable decision support systems.

**Systematic Review Registration:**

https://www.crd.york.ac.uk/PROSPERO/view/CRD420261322606, identifier CRD420261322606.

## 1 Introduction

The International Association for the Study of Pain defines pain as “an unpleasant sensory and emotional experience associated with actual or potential tissue damage, or both” (1). Oro-facial pain (OFP) is a group of conditions that are highly prevalent and clinically important, as they have a significant impact on quality of life and daily functioning (2). These disorders have a wide range of etiologies including dental, musculoskeletal, neurological and adjacent anatomical structures and often manifest with overlapping symptoms and distinctive clinical presentations (3).

OFP is most often caused by odontogenic conditions, which include conditions of dental hard tissues, pulpal tissues, periodontal structures, and supporting bone (4). Pain may result from dentin hypersensitivity, pulpal inflammation, apical periodontitis, periodontal disease, or postoperative complications after dental procedures (2–7). In addition to odontogenic causes, OFP presentations may also be resulting from temporomandibular disorders (TMD), maxillofacial trauma, salivary gland diseases, sinus related conditions, neuralgias, cysts, tumours and neuropathic disorders (8–10). The clinical manifestations overlap considerably and pain perception is multifactorial, which often makes diagnosis difficult and can lead to delayed treatment, misdiagnosis or inappropriate management (11). Moreover, clinical diagnostic errors in pain conditions associated with dentistry are among the most frequently reported adverse clinical events by patients and clinicians (12, 13).

Diagnosis and prediction of orofacial pain is inherently difficult. Pain is not merely a result of structural pathology but is also influenced by complex biological, neurological, psychological and contextual factors. Traditional diagnostic procedures are often based on clinician experience, subjective interpretation of clinical symptoms, and variable clinical findings, which may lead to inconsistencies in diagnosis and treatment planning (11).

AI encompasses a broad range of computational techniques that enable computers to recognize patterns, learn from data, and support clinical decision-making (14). Machine learning (ML), a subfield of AI, involves algorithms that learn predictive relationships from structured data without explicit programming. Deep learning (DL), a subset of ML, utilizes multilayer artificial neural networks capable of automatically extracting complex features, particularly from imaging data. Convolutional neural networks (CNNs) are a class of DL architectures commonly employed for image analysis, while artificial neural networks (ANNs) are frequently applied to structured clinical datasets (14). This review also discusses several other methods, including natural language processing (NLP) for extracting information from clinical text, Bayesian inference models for estimating diagnostic probabilities, and large language models (LLMs) for generating clinically relevant responses from textual information. Understanding these AI methodologies is essential for interpreting their diagnostic capabilities and limitations in OFP.

Over the last decade, advances in machine learning (ML) and deep learning (DL) have increasingly influenced clinical decision making in several domains of healthcare. These technologies are now being investigated for applications like automated pattern recognition, predictive modeling, and imaging interpretation (15). Convolutional neural networks (CNNs) and other artificial intelligence (AI) algorithms have shown moderate to high performance in medical imaging, disease classification, prognostic prediction, and personalized treatment planning in various medical and dental disciplines. The applications of AI in dentistry have been rapidly expanded in orthodontics (16, 17), endodontics (18, 19), prosthodontics (20), restorative dentistry (21), periodontics (22), and oral and maxillofacial surgery (23, 24). These systems have shown the capacity to improve diagnostic accuracy, to enhance workflow efficiency, to assist clinical decision making and to minimize human error (25, 26).

More recently, AI-based approaches have been explored for diagnosis, classification, localization, and prediction of various OFP conditions using various data modalities, including clinical records, imaging, thermography, questionnaires, neuroimaging, and electronic health records (27). However, compared to traditional disease detection tasks, diagnosis and prediction of pain pose particular challenges as the severity of pain and patient-reported symptoms may not necessarily correlate with objective clinical or radiographic findings. Moreover, there is wide heterogeneity in published studies concerning AI architectures, datasets, validation protocols, and performance metrics, which limits direct comparison and clinical application of findings (27, 28).

While the applications of AI in dentistry have been growing exponentially, no systematic review has synthesized the evidence specific to AI-assisted diagnosis and prediction for OFP conditions. Therefore, this systematic review aims to critically assess the current applications, diagnostic performance, methodological quality, and clinical applicability of AI-based systems in diagnosing and predicting OFP disorders.

## 2 Materials and methods

This systematic review was conducted in accordance with the Preferred Reporting Items for Systematic Reviews and Meta-Analyses of Diagnostic Test Accuracy Studies (PRISMA-DTA) guidelines (29) the details are presented in Supplementary Table S1. The review protocol was prospectively registered in the International Prospective Register of Systematic Reviews (PROSPERO) under registration number CRD420261322606. The review question and eligibility criteria were formulated using the PICO framework (Population, Intervention, Comparison, and Outcome), as presented in Table 1.

**Table 1:** Description of the PICO (P = population, I = intervention, C = comparison, O = outcome) elements.

Research question	What are the diagnostic and predictive performances of artificial intelligence–based systems in the assessment and management of Oro-facial pain conditions?
Population	Patients presenting with Oro-facial pain conditions include those experiencing general orofacial pain, temporomandibular disorders (TMDs), trigeminal neuralgia, facial pain syndromes, postoperative odontogenic pain, neuropathic pain, and dental pain.
Intervention	Artificial intelligence–based diagnostic and predictive approaches include machine learning (ML), deep learning (DL), artificial neural networks (ANNs), convolutional neural networks (CNNs), large language models (LLMs), and other AI-assisted clinical decision support systems.
Comparison	Conventional clinical diagnostic approaches include expert clinician assessments, statistical prediction models, traditional diagnostic methods, non-AI computational systems, and internally validated reference standards.
Outcome	Diagnostic accuracy, predictive performance, classification capability, sensitivity, specificity, area under the curve (AUC), model validation, and clinical applicability of AI systems are critical evaluation metrics.

### 2.1 Search strategy

A systematic electronic literature search was performed in PubMed, Scopus, Embase, Cochrane Library, Web of Science, and Google Scholar to find relevant studies published from January 1, 2000, to February 1, 2026. The literature search was conducted for articles published from January 2000 to February 2026 to comprehensively cover the evolution of artificial intelligence techniques applied to OFP. Although contemporary deep learning models have become prevalent over the past decade, earlier studies using artificial neural networks, Bayesian inference systems, and traditional machine learning algorithms established the methodological foundation for current AI-assisted diagnostic systems and were therefore included.

The search strategy included controlled vocabulary terms (MeSH terms) and keywords related to artificial intelligence and OFP conditions. The main search terms were: artificial intelligence, machine learning, deep learning, artificial neural networks, convolutional neural networks, automated models, oral pain, orofacial pain, oro-maxillofacial pain, neuropathic pain, temporomandibular disorder pain, diagnosis, prediction, classification. The search strategy was optimized using the boolean operators “AND” and “OR”. Full, database-specific search strategies are provided in Supplementary Table S2.

A simple combination of keywords was used to search Google Scholar: “artificial intelligence” AND “oral pain” AND “oro-maxillofacial pain.” We maintained methodological consistency and relevance by screening only the first 200 results sorted by relevance, as studies beyond this threshold were significantly less relevant and there was an increased likelihood of duplication.

Furthermore, a manual search of the reference lists of eligible studies and relevant review articles was undertaken to identify potentially missed publications. All retrieved records were imported into reference management software and duplicates were removed.

### 2.2 Study selection

We identified 302 records in total; 300 were identified from electronic database searching and two from manual searching. Following the removal of 210 duplicates, 92 articles were available for title and abstract screening. Two independent reviewers (S.B.K and A.A.) screened all records using predefined eligibility criteria. Any disagreements that arose during the screening process were resolved through discussion and consensus, with consultation from a third reviewer when necessary (G.C.O).

**Figure 1:**
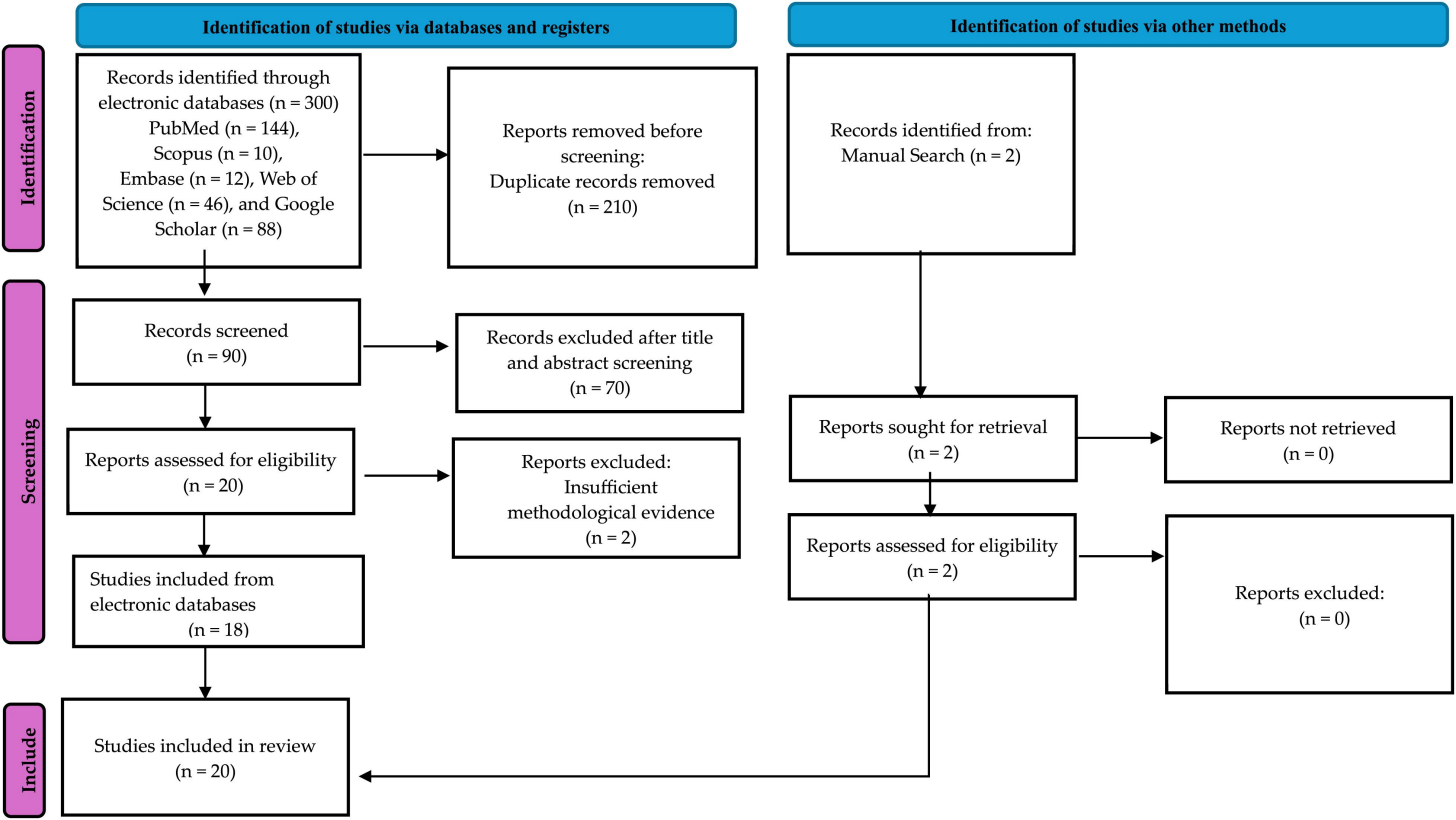
PRISMA 2020 flow diagram for new systematic reviews which included searches of databases, registers and other sources.

Full-text articles were assessed for eligibility after title and abstract screening. Twenty studies that met the inclusion criteria were included in the qualitative synthesis. Figure 1 shows the PRISMA flow diagram of the study selection process.

### 2.3 Criteria for inclusion and exclusion

Eligible studies were those that satisfied the following criteria: (a) were original research papers that investigated AI-based technologies. (b) evaluated AI applications for the diagnosis, classification, localization, or prediction of oro-facial pain conditions. (c) quantitative outcome measures, such as accuracy, sensitivity, specificity, AUC, or other predictive performance metrics; and (d) sufficient methodological details on the development, validation, or testing of the AI model. No restrictions were imposed on study design. Exclusion criteria were: narrative reviews, conference abstracts without full-text, editorials, unpublished studies, studies not related to AI applications, and articles in languages other than English.

### 2.4 Extraction of data and qualitative synthesis

Data extraction was performed independently by two reviewers (F.H and B.A) using a standardized data extraction form. The extracted variables included author information, year of publication, study aims, type of AI model, details of the dataset, validation methods, outcome measures, diagnostic or predictive performance metrics, and key findings as reported in Table 2.

**Table 2:** Qualitative synthesis of included studies.

SlNo	Authors	Year of publication	Study design	Algorithm architecture	Objective of the study	No. of patients’/images/photographs for testing	Study factor	Modality	Comparison if any	Evaluation accuracy/average accuracy/statistical significance	Results (+) effective (−) non effective (N) neutral	Outcomes	Authors suggestions/conclusions
1.	Clark et al. (32)	2025	Retrospective data with prospective validation	NBI (Naïve Bayesian Inference)	To develop an AI based tool to assist diagnosis of orofacial pain	1,020 patient data sets	Orofacial pain	Clinical data- examination and history	Internal comparison	Mean predicted probability- 84.25%	Effective	NBI outperformed all 5 ML models (DT, RF, GB, SVM, NN) in the diagnosis of orofacial pain	AI based diagnostic tool can be used a support tool for clinical decision making
2.	Burchiel et al. (33)	2025	Observational study	MLbased diagnostic decision support system	To develop a ML based model to diagnose TMDs or Trigeminal neuralgias	101 patients	Orofacial pain-TMDs or TNs	Clinical data	Comparison among models (Random Forest and SVM) & with expert opinions	Random forest- showed best F1 score: 0.953, ROC: AUC-0.952; Overall Diagnostic accuracy-90%	Effective	Random Forest performed best among the models in diagnosis of TMDs and TNs	AI diagnostic tools are feasible and can assist clinicians
3.	Nocera et al. (34)	2021	Observational study from retrospective data	ML models	To develop and evaluate a diagnostic automated system for orofacial pain	451 patient cases	Orofacial pain disorders	Clinical data and questionnaire	Compared Models with expert-based models	Accuracy:75.6-96.89%; Precision:52.40%-97.38%. Recall: 46.27%–98.21%; F-1 score: 0.56–0.96.	Effective	ML models showed high diagnostic accuracy; SVM and RF performance was best	ML based diagnostic tools are feasible and can support clinical decision making
4.	Kreiner et al. (35)	2022	Experimental study	ANN, MLP	To develop an ANN based diagnostic model for orofacial pain and TMD	11 Cases	Orofacial pain, TMD, dental pain, neuropathic, Trigeminal neuralgia	Clinical cases	ANN vs. 12 general dental clinicians	ANN more accurate (*p* < 0.05) than clinician; more accuracy in non-dental/referred pains	Effective	ANN outperformed clinicians for non-dental pains, comparable performance for dental pain	ANN can assist clinicians in accurate diagnosis
5.	Umapathy et al. (36)	2021	Prospective e study	ML, DL	To develop an automated system using thermography & ML/DL for detection of orofacial pain	100 subjects	Orofacial pain	Facial thermograms	Internal comparison (ML vs. DL)	Accuracy: SVM-99%, RF-99%, VGG-97%, DenseNet-68%; Sensitivity: 100%	Effective	ML (SVM, RF) performed better than DL; Thermography shows strong discriminatory power for orofacial pain detection	Thermography + ML act as a non-invasive screening and diagnostic aid
6.	Nam et al. (37)	2018	Retrospective study	NLP + Decision Tree	To differentiate TMD mimicking conditions from genuine TMD pain using AI based analysis of clinical text and examination data	319 patients	TMD and TMD mimicking conditions	EHR clinical text and clinical data	Comparing TMD patients and TMD mimicking patients (internal comparison)	Accuracy: 96.6%, Sensitivity: 69%, Specificity: 99.3%	Effective	AI based models can differentiate both TMD and TMD mimicking conditions separately	NLP and Decision Tree can aid in differential diagnosis and clinical decision making
7.	Zatt et al. (38)	2024	Retrospective	Machine Learning (D T)	To assess a ML model to classify TMD based on ICOP 1	122 medical records	TMD joint pain; Myofascial pain	Clinical records	Internal comparison	TMD-Accuracy: 84%; weighted avg-78%; myofascial pain-accuracy: 78%; weighted avg: 85%	Effective	ML model can classify TMD conditions with good accuracy	AI can be used as a complementary tool to support diagnosis
8.	Xu et al. (39)	2026	Retrospective study	Multiple ML models	To develop and interpret ML models based on MRI characteristics.	584 patients, 755 TMJ MRI data sets	TMD pain	MRI based imaging	Internal comparison	Light GBM model AUC of 0.899, Sensitivity: 0.798, specificity:0.832, Accuracy: 0.815	Effective	MRI based ML models can predict the TMD pain severity Light GBM was found to be better among all (LR, SVM, RF, XG Boost, AdaBoost, GBC, BC, ETC, DTC, ML)	MRI-based ML model with SHAP analysis may help identify high-risk TMD patients
9.	Gao et al. (40)	2021	Prospective study	ANN	To evaluate the accuracy of ANN model for predicting postoperative pain following RCT.	300 patients	Prediction of post-operative pain after RCT	Clinical data	No comparison (only internal validation of ANN)	Training accuracy of training samples: 95.2%	Effective	ANN showed high predictive accuracy	ANN is useful for predicting pain and aiding treatment planning
10.	Yadalam et al. (41)	2022	Prospective observational study	AI based multiple linear regression models (MLR)	To predict post-operative pain after dental implant surgery	1,032 patients	Prediction of post-operative after dental implant surgery	Clinical data	No comparison as such, model performance is evaluated	MLR—Accuracy: 89.6. Root means square deviation: 0.1085	Effective	MLR model predicts pain accurately with good predictive capability	AI prediction is feasible clinically
11.	Freitas et al. (42)	2026	Secondary analysis of prospective data	ML models	To develop and validate the ML models in predicting post operative pain after RCT	354 patients	Prediction of post-operative endo pain after irreversible pulpitis	Clinical data	Internal comparison	For 72 h-SVM is best-AUC-0.81; Precision-0.88	Effective	SVM performed best among the ML models(RF, GB, DT, KNN)	ML can support personalized pain prediction and can be integrated into clinical practice
12.	Neychev et al. (43)	2024	Randomized Controlled Trial-split mouth study	Deep Learning-Neural network	To develop an objective DL based model to assess the effect of preemptive analgesia on postsurgical pain	40 patients (80 sample for model training)	Prediction of postsurgical pain after third molar extraction	Clinical data-non imaging	DL model and statistical methods	Model accuracy: 92.6%, *p* = 0.007 for pain at 6th hr.	Effective	Model can predict postoperative pain levels and analgesic needs; preemptive analgesic shows positive effect	The use of DL enables the prediction of pain intensity and the need for an additional dose of analgesics
13.	Yu et al. (44)	2023	Prospective study	ML algorithms (RF, LDA, XG Boost, SVM, Gaussian NB, LR, DT, AdaBoost, KNN)	To develop a prediction model based on ML algorithms to identify patients at high risk of postoperative pain after tooth extraction.	185 patients, 202 cases	Prediction of post-operative pain after tooth extraction.	Radiographic images	Internal comparison	Random forest –The AUC, sensitivity, specificity, and accuracy were 0.879 (0.861–0.891), 0.857, 0.846, and 0.848, respectively-for internal validation set	Effective	ML model can identify patients at high risk of severe postoperative pain; surgeon's qualification, pain sensitivity, BMI, BP and OHI-S are key predictors for this model	This study developed a machine learning-based postoperative pain risk prediction model for impacted mandibular third molar extraction.
14.	Salama et al. (45)	2025	Retrospective cohort study	SVM, RF, GBM	To predict acute pain severity and opioid doses during RT using ML models (LR vs. SVM, RF vs. GBM)	900 patients	Prediction of odontogenic pain	Clinical and EHR data	Internal comparison	Pain prediction-GBM-AUROC = 0.71; Analgesic efficacy-RF/GBM-AUROC = 0.68	Effective	ML models are accurate moderately in predicting pain, opioid use and analgesic response	ML model showed promise for risk stratification and personalized pain management
15.	Limonadi et al. (46)	2006	Prospective study	ANN	To evaluate an ANN to diagnose facial pain syndrome based on questionnaires	143	Facial pain syndrome	Questionnaire based	Experts’ opinion	Accuracy 95% (95/100). Sensitivity: 84% (TN1). Specificity: 83% (TN1)	Effective	ANN can diagnose facial pain syndrome especially Trigeminal neuralgia based on patient's history	ANN showed feasibility in assessing facial pain; however, due to the limited sample size performance might be limited
16.	McCartney et al. (10)	2014	Prospective study	ANN	To develop and validate ANN based system to diagnose facial pain syndromes	607 patients	Facial pain syndromes	Clinical data-questionnaire based	Expert clinician diagnosis	Overall agreement-accuracy:87.1%; TN1—Sensitivity-92.4%, Specificity-87.8%, TNP-Sensitivity-86.7%, Specificity-95.2%, STN, PHN-100% (Sensitivity, Specificity)	Effective	ANN demonstrated high diagnostic accuracy	ANN based questionnaire system enables accurate self-diagnosis and clinical decision support
17.	Suresh et al. (47)	2024	Retrospective observational	ML	To predict the need for anticonvulsant drugs in patients with orofacial neuropathic pain	178 patients	Facial pain syndromes	Clinical data	Internal comparison	Accuracy-Adaboost-97%, Decision tree-94%, Random forest-89%; AUC-0.97, 9.94, 0.897	Effective	ML showed high predictive accuracy; Adaboost-performed best	ML can assist clinicians in treatment decision tree
18.	Kim et al. (48)	2009	Cross sectional study	ANN	To predict dental pain using neural network based on eating habits and recognition factors of people	NR	Dental pain	Questionnaire based (non-imaging)	Not reported	Model fitness Accuracy-80%	Effective	Neural network can predict likelihood of dental pain	Model suggested that factors such as preventive measures including proper eating habits, education on oral hygiene, and stress release should be implemented before a dental treatment
19.	Chattopadhyay et al. (49)	2012	Observational study	NBC, LBC	To develop a Bayesian classifier for diagnosing dental diseases using pain parameters	40 cases for 10 dentists (40*10 = 400 case evaluations)	Dental pain	Questionnaire based-clinical data	No direct comparison, only validation with dataset	Avg diagnostic Accuracy: 72%.	Effective	While using pain parameters, Bayesian approach showed moderate diagnostic accuracy; LBC showed improved performance over NBC	Bayesian approach is useful for clinical decision making
20.	Hu et al. (50)	2019	Experimental study	ANN	To develop a real time AI based augmented reality framework for objective pain detection and localization from brain activity	21 patients	Acute dental pain	Neuroimaging data	Internal comparison NN models-ANN vs. CNN, RNN, LSTM	Pain detection-Accuracy = 80.37% (ANN), Sensitivity: 0.326, Specificity:0.861. Pain localization Accuracy = 74.23%(CNN)	Effective	ANN was best for pain detection, CNN was best for pain localization,	Real time objective pain assessment need optimization, more dataset and validation for clinical use

SVM, support vector machine; RF, random forest; XGBoost, extreme gradient boosting; LightGBM, light gradient boosting machine; AdaBoost, adaptive boosting; GBC, gradient boosting classifier; BC, bagging classifier; ETC, extremely randomised trees; DTC, decision tree classifier; MLP, multi-layer perception; AUC, receiver operating characteristic curve; BMI, body mass index; OHI-S, simplified oral hygiene index; BP, blood pressure; LDA, linear discriminant analysis; Gaussian NB, Gaussian Naive Bayes; KNN, K-nearest neighbors; DT, decision tree; AI, artificial intelligence; TN 1, trigeminal neuralgia type1; ICOP, international classification of orofacial pain; NBI, Naïve Bayesian inference; DT, decision tree; GB, Gradient boosting; NN, neural network; AUC, area under the curve; MLP, multi layer perceptron; MLR, multiple linear regression; RCT, root canal treatment; TNP, trigeminal neuropathic pain; STN, symptomatic trigeminal neuralgia; PHN, post herpetic neuralgia; NBC, Naïve Bayesian classifier; LBC, learned Bayes’ classifier; EHR, electronic health record; GBM, gradient boosting machine; AUROC, area under the receiver operating curve; CNN, convolutional neural network; RNN, recurrent neural network; LSTM, long short-term memory; NLP, natural language processing; NR, not reported by the original study authors.

Journal names and author identifiers were masked prior to data evaluation to reduce possible bias by reviewers in data evaluation. Inter-reviewer reliability for study selection and data extraction was substantial with a Cohen's kappa coefficient of 88%.

Given the high heterogeneity in the AI architectures, datasets, imaging modalities, outcome measures, validation strategies and reporting methods, we could not perform a quantitative meta-analysis. So a qualitative narrative synthesis was performed.

### 2.5 Evaluation of methodological quality and risk of bias

Two reviewers (K.I and V.K) independently evaluated the methodological quality and risk of bias of the included studies using the Quality Assessment of Diagnostic Accuracy Studies 2 (QUADAS-2) tool (30). QUADAS-2 assesses four domains: patient selection; index test; reference standard; and flow and timing. Risk of bias was assessed in each domain. The first 3 domains were also assessed for applicability concerns. Discrepancies between reviewers were solved by discussion and consensus. Inter-reviewer agreement for QUADAS-2 assessment was excellent (Kappa Cohen's *κ* = 0.88).

The majority of included studies had a low risk of bias for patient selection and index test domains due to the use of well-defined clinical datasets and standardized AI training procedures. However, several studies had a moderate to high risk of bias in the reference standard domain due to insufficient comparator standards, lack of external validation and reliance on internal model-based validation approaches. There also existed concerns for applicability in studies with institution specific data and non-standardized diagnostic criteria.

**Figure 2:**
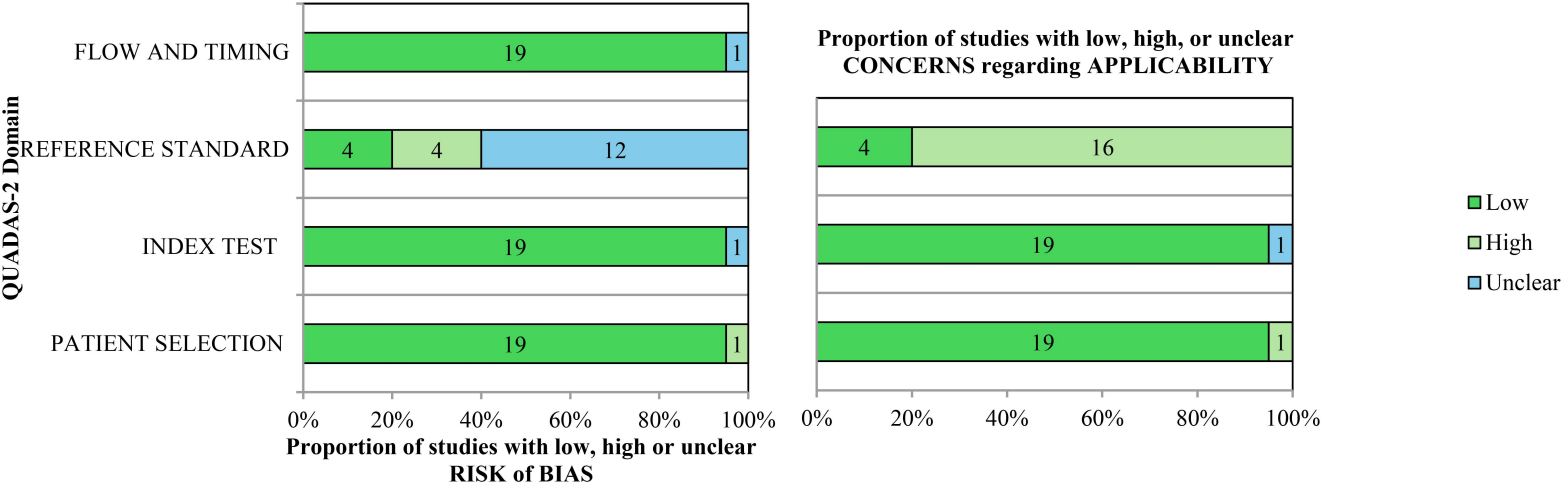
QUADAS-2 assessment of the individual risk of bias domains and applicability concerns.

The QUADAS-2 results are summarized in Figure 2 and Supplementary Table S3 to improve the interpretability and transparency of the quality assessment.

### 2.6 Certainty of the evidence assessment

The certainty of evidence for the included studies was evaluated using the Grading of Recommendations Assessment, Development, and Evaluation (GRADE) framework (31). The certainty of evidence was graded as high, moderate, low, or very low according to the evaluation of five domains: risk of bias, inconsistency, indirectness, imprecision, and publication bias.

The overall certainty of the evidence was graded as moderate for most of the outcomes. The main reasons for downgrading of the certainty of evidence were methodological heterogeneity across studies, retrospective study designs, inconsistent reporting of validation procedures, minimal external validation, and heterogeneity of outcome measures and AI architectures. Use of institution specific datasets and differences in diagnostic reference standards were also raised as concerns regarding indirectness. Imprecision: Studies with small sample sizes and inadequate reporting of confidence intervals or calibration metrics were imprecise.

We rated the certainty of evidence for AI applications in dental pain localization and classification as low, due to significant heterogeneity, methodological limitations and higher risk of bias. The detailed GRADE assessment is provided in Table 3.

**Table 3:** Assessment of strength of evidence.

Outcome	Inconsistency	Indirectness	Imprecision	Risk of bias	Publication bias	Strength of evidence
Application of AI in diagnosing general orofacial pain (32–36)	Not Present	Not Present	Not Present	Present	Not Present	⊕⊕⊕◯
Application of AI in diagnosing TMD and TMJ pain (37–39)	Not Present	Not Present	Not Present	Present	Not Present	⊕⊕⊕◯
Application of AI in prediction of postoperative odontogenic pain (40–45)	Not Present	Not Present	Not Present	Present	Not Present	⊕⊕⊕◯
Application of AI in diagnosing facial pain syndromes including trigeminal neuralgia (10, 46, 47)	Not Present	Not Present	Not Present	Present	Not Present	⊕⊕⊕◯
Application of AI in diagnosis and localization of dental pain (48–50)	Present	Present	Present	Present	Not Present	⊕⊕◯◯

⊕⊕⊕⊕, high evidence; ⊕⊕⊕◯, moderate evidence; ⊕⊕◯◯, low evidence; ⊕◯◯◯, very low evidence.

## 3 Results

### 3.1 Selection of studies and their characteristics

Qualitative synthesis included twenty studies that fulfilled the inclusion criteria. The studies included in this review were published between 2006 and 2026 and demonstrated a steady increase in the use of AI for diagnosis and prediction of OFP pain conditions in the last decade. Most studies were retrospective or observational in nature, with a smaller number being prospective or experimental. The studies investigated diverse AI techniques like machine learning (ML), deep learning (DL), artificial neural networks (ANN), convolutional neural networks (CNN), natural language processing (NLP), and Bayesian inference systems.

The main clinical applications identified in the studies are the diagnosis of general orofacial pain (32–36), classification of temporomandibular disorders (TMDs) (37–39), prediction of postoperative odontogenic pain (40–45), diagnosis of trigeminal neuralgia and facial pain syndromes (10, 46, 47), localization and prediction of dental pain (48–50). The qualitative synthesis of included studies is presented in detail in Table 2.

During data extraction, the diagnostic reference standard used in each study was recorded to facilitate the interpretation of AI model performance across various pain conditions. The details are presented in Supplementary Table S4.

### 3.2 A comparison of AI modality performance

Included studies were found to have significant heterogeneity of the AI architectures, input datasets, validation approaches and outcome measures. However, several consistent performance patterns were identified across AI modalities and clinical applications.

In general, ML based models showed consistently high diagnostic and predictive performance, particularly when structured clinical datasets or imaging-based modalities were used. Random Forest (RF), Support Vector Machine (SVM) and Gradient Boosting algorithms have often shown better performance than other AI approaches in various clinical domains.

Deep learning models have shown promising performance in imaging intensive applications, like thermography, MRI based prediction and neuroimaging analysis. Deep learning models, however, require larger datasets and show more variation in performance than traditional ML algorithms.3.3 Performance Based on Data Modality.

#### 3.2.1 Structured clinical data and questionnaire data

AI models with structured clinical data and variables derived from questionnaires showed consistently favourable diagnostic performance in a variety of orofacial pain conditions. The ML algorithms with clinical variables had diagnostic accuracies of 75%–97%. Of these, the Naïve Bayesian Inference model developed by Clark et al. demonstrated a high level of agreement with expert diagnosis with a mean predicted probability of 84.25% (32). Similarly, Nocera et al. achieved accuracies ranging from 75.6% to 96.89%. Random Forest (RF) and Support Vector Machine (SVM) algorithms showed the best overall performance (33).

ANN systems based on questionnaires have also shown considerable diagnostic potential for facial pain syndromes. Limonadi et al. reported a 95% overall diagnostic accuracy for facial pain disorders using ANN-based questionnaires (46). McCartney et al. reported an overall agreement accuracy of 87.1% with expert diagnoses for trigeminal neuralgia and neuropathic pain syndromes (10). On the contrary, Bayesian classifier methods for dental pain had relatively poor performance. Chattopadhyay et al. found moderate diagnostic accuracy of 72% using Bayesian classification models based on pain parameter (49).

#### 3.2.2 Artificial intelligence models based on imaging

Among the included studies, imaging-based models generally demonstrated better diagnostic metrics than questionnaire-based systems, although performance varied with dataset size and validation strategy. These models were mainly based on thermography, MRI and other neuro-imaging modalities.

The highest diagnostic performance reported in this review was by Umapathy et al., where thermography-based ML models achieved accuracies of up to 99% using SVM and RF classifiers (36). Interestingly, conventional ML algorithms outperformed DL architectures like VGG-16 and DenseNet, indicating that traditional ML methods may be more suitable for interpreting structured thermal features in smaller datasets.

MRI-based ML models have also demonstrated good predictive power in TMD. Xu et al. obtained an area under the curve (AUC) of 0.899, sensitivity of 0.798, specificity of 0.832, and total accuracy of 0.815 for the LightGBM model in the prediction of TMD pain severity (39). Further, the incorporation of SHAP-based interpretability analysis improved the transparency and clinical applicability of the model.

Neuroimaging-based AI systems have shown moderate but promising performance in objective pain localization. Hu et al. developed a real-time augmented reality framework using ANN and CNN architectures to detect and localize from brain activity with 80.37% accuracy in pain detection and 74.23% accuracy in pain localization (50).

### 3.3 Performance of AI in selected clinical applications

#### 3.3.1 Diagnosis of orofacial pain

Five studies examined AI applications in diagnosing general orofacial pain conditions (32–35). Diagnostic performance varied with the data modality and AI architecture used. ML models based on structured clinical datasets showed good diagnostic accuracy, especially RF and SVM algorithms (32, 34). AI systems based on thermography had the highest discriminatory capacity among all the studies included (36). These findings suggest that AI systems can be useful adjuncts in clinical decision support, particularly for non-specialist clinicians and for initial screening purposes.

#### 3.3.2 Temporomandibular disorders (TMDs) and temporomandibular joint (TMJ) pain

AI applications for TMD diagnosis have shown consistently outstanding performance on structured clinical datasets and imaging-based models. Nam et al. (37) applied natural language processing (NLP) and decision tree models to differentiate TMD from TMD-mimicking conditions with a diagnostic accuracy of 96.6% and a specificity of 99.3%. In a similar way, Zatt et al. (38) published the use of ML decision tree models based on the International Classification of Orofacial Pain (ICOP) criteria, where the accuracy was 84% for the classification of TMJ pain and 78% for the classification of myofascial pain.

Comparative ML studies show that RF models are superior across the board to other algorithms. Burchiel et al. reported the RF model accuracy of 90% with Receiver Operating Characteristic—Area Under the Curve (ROC-AUC) of 0.952 for differentiating TMD from trigeminal neuralgia (33). In summary, models based on imaging MRI and structured clinical data showed comparable diagnostic potential but MRI-based systems offered better objective assessment and risk stratification.

#### 3.3.3 Prediction of the postoperative odontogenic pain

Six studies have explored AI models for predicting postoperative odontogenic pain in patients undergoing endodontic treatment, implant placement, tooth extraction, and oral surgery (40–45). ANN-based prediction systems have been demonstrated to achieve especially high predictive accuracy. Gao et al. (40) used back propagation ANN models to predict post-operative pain after root canal treatment and reported a predictive accuracy of more than 95%. Similarly, Neychev et al. reported 92.6% accuracy for prediction of postoperative pain after extraction of third molars using DL (43).

Among the ML algorithms, the RF and SVM models performed the best in terms of predictive performance. Yu et al. reported an RF model with AUC of 0.879, sensitivity of 0.857 and specificity of 0.846 to predict postoperative pain after mandibular third molar extraction (44). SVM was the best model to predict post-endodontic pain with an AUC of 0.81, according to Freitas et al. (42).

Most AI models showed good predictive performance, however, studies to predict cancer-related pain showed relatively moderate results with the Area Under the Receiver Operating Characteristic (AUROC) value around 0.71 (45).

#### 3.3.4 Trigeminal neuralgia and facial pain syndromes

AI systems have demonstrated significant diagnostic ability for trigeminal neuralgia and neuropathic facial pain syndromes.

ANN-based models have been shown to be highly sensitive and specific in the diagnosis of trigeminal neuralgia. McCartney et al. (10) reported sensitivity of 92.4% and specificity of 87.8% for trigeminal neuralgia type 1. Likewise Limonadi et al. reported a total diagnostic accuracy of 95% for facial pain syndromes using ANN-based questionnaires (46).

In neuropathic pain, ML models have also performed well in the prediction of treatment-related outcomes. Prediction of anticonvulsant treatment—predictive accuracies ranging from 89% to 97% reported AdaBoost being the best performer (47).

However, AI performance was reduced in less common or clinically complex pain syndromes, suggesting limitations in generalizability and dataset representation.

### 3.4 Comparative interpretation: machine learning vs. deep learning vs. artificial intelligence vs. clinicians

In most of the included studies, conventional ML models outperformed DL approaches, especially in studies using smaller structured datasets. Random forest, support vector machines (SVM) and Bayesian inference models were all more accurate, interpretable and robust than the more computationally intensive DL architectures.

DL models have greater utility in imaging-based applications like MRI, thermography and neuroimaging, where hierarchical feature extraction offers significant advantages. However, DL models are often more variable in performance and appear to be heavily dependent on dataset size and image quality.

Direct comparison of AI performance to clinicians showed that AI systems generally performed as well as nonexpert practitioners but less effectively than expert clinicians in complex diagnostic scenarios. Overall, AI models showed the most clinical utility as adjunctive decision-support tools, not as independent diagnostic replacements.

### 3.5 Risk of bias and certainty of evidence

The QUADAS-2 (30) assessment of methodological quality revealed a low risk of bias in the patient selection and index test domains in general. Most of the research used patient-derived clinical data as input for different AI models, such as electronic health records, questionnaires, imaging modalities (e.g., MRI, radiography), and other diagnostic data sets. The patient selection domain was rated as having a low risk of bias, given that most of the studies used real-world clinical data with well-defined inclusion criteria.

However, the study by Kim et al. (48), had an insufficient description of sample size and methodology, leading to a high risk of bias in the patient selection and applicability domains. In the reference standard domain, the absence of an explicit comparator or external reference standard further increased the level of applicability concerns. The index test domain had a low risk of bias due to the consistent use of machine learning and deep learning algorithms, with clear training and validation techniques. However, there were some issues with reproducibility due to the different architectures of the models employed. In some of the included studies, the reference standard used to analyse the results of the index test was not described properly, raising concerns about potential reference standard and applicability domain bias. Chattopadhyay et al. (49), Gao et al. (40) and Yadalam et al. (41) did not have a proper comparator or reference standard in their research, and their analyses were mainly limited to internal dataset validation and model-based evaluation. Similarly, several other studies including Xu et al. (39), Neychev et al. (43), Yu et al. (44), Zatt et al. (38), Freitas et al. (42), Nam et al. (37), Clark et al. (32), Suresh et al. (47), Umapathy et al. (36), Salama et al. (45), and Hu et al. (50) were mostly based on internal comparison among different AI models and lacked an external reference standard. One of the selected studies, by Kreiner et al. (35), had reference standard based on observations by general dentistry practitioners, so it is likely that variability of results may exist due to differences in the levels of clinical experience of the clinicians.

Therefore, about 20% of the studies used in the analysis had high risk of bias in the reference standard domain, especially for the assessment of the reference comparator and the validation process. In contrast, 80% of the studies had high applicability concerns in the reference standard domain. In general, low risk of bias was found in patient selection, index test and flow and timing domains in most of the studies but substantial applicability concerns were seen in the reference standard domain. The risk of bias and applicability assessment of the included studies are illustrated in Figure 2.

The grading of evidence certainty in this systematic review was done using the Grading of Recommendations Assessment Development and Evaluation (GRADE) approach (31). Evidence certainty was rated as very low, low, moderate and high based on five main domains including publication bias, indirectness, imprecision, risk of bias and inconsistency. Most of the outcome categories presented moderate certainty of evidence according to the analysis, while the use of AI for diagnosis and localization of dental pain was classified as having a low level of certainty of evidence due to methodological heterogeneity, inconsistent reporting, indirectness, imprecision, and increased risk of bias. Overall, the certainty of evidence for the application and performance of AI in diagnosing and predicting OFP was moderate. The details are presented in Table 3.

## 4 Discussion

This systematic review evaluated the current evidence on the utilization of AI in the diagnosis, classification, localization and prediction of OFP conditions. Overall, the results suggest that AI-based systems can assist clinicians in specific diagnostic tasks to improve diagnosis accuracy, risk stratification and clinical decision-making in complex pain disorders. ML algorithms, especially random forest (RF), support vector machine (SVM) and Bayesian inference models, consistently showed favorable performance across structured clinical datasets, whereas DL models seemed especially helpful in imaging-intensive applications, such as thermography, MRI analysis and neuroimaging-based pain localization.

Despite these promising results, the current evidence base continues to be methodologically heterogeneous and clinically immature. The included studies varied in study design, dataset characteristics, validation strategies, reference standards, and outcome measures, which limits direct comparison between studies and reduces the overall generalizability of the findings. AI promises a lot regarding OFP diagnostics, but its implementation in routine clinical practice is yet to be considered timely.

### 4.1 AI modalities comparison

One of the important findings of this review was that conventional ML algorithms still perform better than more computationally intensive DL algorithms. Several studies have shown RF, SVM and Bayesian inference models to have high diagnostic accuracy and good predictive capability, particularly when trained on structured clinical datasets and variables derived from questionnaires. These results would indicate that the structured datasets curated by clinicians might now be more reliable in terms of discriminatory features compared to the relatively smaller imaging datasets typically used in dental AI studies.

Interestingly, several studies showed that traditional ML algorithms outperform DL architectures for thermography-based OFP detection (36). This observation might be attributed to the small size of many dental AI datasets as DL models often need much larger datasets to have stable and generalizable performance. Conventional ML algorithms, on the other hand, are less data-driven and may therefore be more appropriate for the smaller clinical datasets often encountered in oral health research.

DL models have been comparatively more useful in imaging-intensive applications, particularly in the prediction of TMD from MRI data and in neuroimaging-guided pain localization (36, 50). The models utilize the capabilities of hierarchical feature extraction, allowing the identification of subtle imaging patterns that might be missed by traditional analysis. A major challenges to reproducibility and external validation is variability in imaging acquisition protocols, annotation methods, and dataset quality.

The performance of several AI algorithms was compared using the same internal dataset in multiple included studies. Such comparisons are useful for assessing the relative performance and consistency of individual algorithms; however, they primarily demonstrate algorithmic convergence rather than true diagnostic accuracy. To robustly evaluate diagnostic performance, comparisons to an independent clinical reference standard and validation with externally derived datasets are necessary. Therefore, the positive results reported by several studies should be interpreted with caution until prospective multicenter external validation is available.

### 4.2 AI systems: explainability, transparency and trustworthiness

One of the main challenges identified across the included studies was the limited explainability and transparency of AI-based diagnostic systems. Although these models may be highly predictive, their lack of interpretability may dramatically compromise clinician trust, clinical adoption, and raise ethical and medico-legal issues.

Explainability is particularly important in clinical pain diagnosis, where diagnostic decisions are often based on multifactorial reasoning that integrates patient history, psychosocial factors, imaging findings, and subjective symptom interpretation. Therefore, clinicians may be hesitant to trust AI predictions without understanding the reasons for these decisions.

So far, only a few studies have incorporated explainable AI approaches. Notably, Xu et al. used SHapley Additive exPlanations (SHAP) analysis to identify MRI-derived predictors of TMD pain severity (39). These approaches enhance the model's transparency by allowing clinicians to interpret the importance of features and the relative contribution of specific variables to AI predictions. Similarly, Local Interpretable Model-agnostic Explanations (LIME) and attention-based visualization techniques can improve interpretability and aid clinician acceptance.

Future AI research in OFP should include the development of explainable and transparent AI frameworks that include clinician-interpretable reasoning pathways in addition to predictive outputs. Clinicians may require some level of interpretability to trust AI generated recommendations for complex pain conditions.

### 4.3 Bias, overfitting and methodological limitations of the dataset

In the present review, we found several methodological limitations that were repeated across the included studies, particularly related to the size of the dataset, external validation, and retrospective study design, which increase the risk of overfitting and selection bias.

The issue of class imbalance was repeatedly mentioned in a number of studies, especially those dealing with rare pain disorders such as trigeminal neuralgia and neuropathic facial pain syndromes. AI models trained on imbalanced datasets often optimize for performance for majority classes, resulting in lower performance on less represented, but clinically important conditions. This constraint can artificially inflate reported accuracy metrics and reduce diagnostic reliability in real-world settings.

In addition, most of the included studies used single-center datasets from very specific clinical populations. Variations in imaging protocols, diagnostic criteria, clinical documentation, and patient demographics across institutions can significantly affect model performance and external validity. This implies that AI models performing effectively on internally validated data may not generalize well to larger and more diverse populations.

The risk of over-fitting remains a major concern in dental AI research. Several studies have reported very high diagnostic accuracies, above 95%, particularly on smaller datasets with limited external validation (36, 40, 47). While these results appear promising, models trained on small homogeneous datasets could eventually give up learning dataset-specific artifacts rather than clinically meaningful disease patterns. This becomes particularly problematic when external validation cohorts are not available.

Furthermore, there was significant heterogeneity with respect to reference standards, validation strategies, performance metrics and reporting quality across studies. Lack of standardized reporting frameworks for AI makes it difficult to make meaningful comparisons between studies and contributes to inconsistencies in evidence synthesis.

### 4.4 External validation and generalizability

A major limitation identified throughout the literature reviewed is the limited generalizability of current AI systems. The majority of the available evidence was based on retrospective single-center datasets, limiting confidence in the clinical generalizability of the models. Only a few studies included prospective validation or external, multicenter testing. Demographic, psychosocial, behavioral and cultural factors have a significant effect on the diagnosis and prediction of OFP. Therefore, AI systems trained on datasets limited to specific geographic locations or institutions might not generalize well to other clinical environments or populations.

While several of these studies report favorable diagnostic performance, many were developed using relatively small datasets. It is important to note that there is no single minimum sample size for AI model development, as data requirements vary depending on the model's complexity, feature dimensionality, class distribution, and intended clinical application. Several hundred well-characterized observations may suffice for conventional machine learning algorithms to achieve stable performance; however, deep learning models generally require much larger datasets to reduce overfitting and improve generalizability. Therefore, the robustness of models should be assessed not only by sample size but also through appropriate external validation, calibration, and reproducibility in independent populations.

In addition, the development of AI models is limited by the absence of standardized diagnostic criteria for orofacial pain conditions. Differences in diagnostic criteria, interpretation of imaging, and pain classification systems are potential sources of heterogeneity for training datasets and lack of reproducibility across studies.

Algorithmic bias and inequitable diagnostic performance may be partly due to the limited inclusion of ethnically and geographically diverse populations in current datasets. Future research should therefore focus on multicenter collaborative datasets, standardized diagnostic frameworks such as the ICOP criteria, and externally validated prospective study designs to improve model robustness and generalizability.

### 4.5 Clinical translation and applicability in the real world

AI has shown promising diagnostic and predictive performance in many studies, however significant challenges remain for realistic integration of these technologies into routine clinical practice.

First, most existing AI systems remain investigational and have not been subjected to rigorous prospective real-world validation. Robust external validation across diverse patient populations, imaging systems and healthcare settings is necessary for clinical deployment to ensure reliability and reproducibility.

Second, workflow integration remains insufficiently addressed in the literature. To provide meaningful clinical utility, AI systems must be integrated into electronic health records (EHRs), radiographic software, and chairside clinical workflows without adding to clinician burden or disrupting patient care processes.

Third, the regulatory and medico-legal barriers remain very high. AI-generated diagnostic outputs give rise to important questions about accountability, liability and informed consent. Who will be accountable for diagnostic errors remains to be seen, whether it is clinicians, healthcare institutions, software developers or AI manufacturers.

Ethical issues related to patient privacy, data security, and algorithmic fairness and transparency are also important. If AI systems are trained on non-representative datasets, they risk inadvertently perpetuating disparities in health care and introducing unintended biases into diagnostic decision-making. Importantly, the findings of this review suggest that AI is presently best suited as an adjunctive clinical decision-support tool, rather than an autonomous replacement for clinician expertise. Human oversight remains critical, especially in complex pain disorders where symptoms are interpreted subjectively and psychosocial factors are multifactorial.

### 4.6 Directions for the future

Efforts should be directed towards the development of large-scale multicenter AI systems, prospectively validated, based on standardized diagnostic criteria, and including patients of different origins. The integration of multimodal AI solutions such as clinical data, imaging, neuroimaging, thermography and patient-reported outcomes can enhance the accuracy of the diagnosis and prediction.

Moreover, explainable AI frameworks such as SHAP and attention-based visualization methods should be incorporated to ensure transparency and clinician trust. Collaborative models can be developed through joint research while protecting patient privacy, overcoming the challenges of sharing data across institutions and improving the quality of multicenter data for research. Ultimately, prospective real-world clinical trials examining workflow integration, clinician acceptance, cost-effectiveness, and patient outcomes are needed before AI systems can be safely and effectively integrated into routine OFP management.

### 4.7 Strengths and limitations

There are several strengths to this review. To the best of our knowledge, this is the first systematic review to comprehensively evaluate the application of AI across the full spectrum of OFP conditions, including temporomandibular disorders, trigeminal neuralgia, neuropathic facial pain, postoperative odontogenic pain, and dental pain. The review was conducted following PRISMA-DTA guidelines. It was based on an extensive search of multiple databases and employed validated methodological quality (QUADAS-2) and certainty of evidence (GRADE) assessments.

However, it is important to acknowledge several limitations. First, there was considerable heterogeneity across the included studies in terms of AI algorithms, datasets, imaging modalities, outcome measures, and validation methods, which did not allow for quantitative meta-analysis. Second, the majority of studies were retrospective and conducted at single institutions, limiting the generalizability of the findings. Third, most AI models have only undergone internal validation, with relatively few studies reporting external or prospective clinical validation. Finally, publication bias and selective reporting cannot be entirely ruled out, as studies demonstrating favorable AI performance may be more likely to be published. These limitations should be considered when interpreting the findings and highlight the need for standardized reporting, multicenter validation, and prospective clinical studies. The limited number of eligible studies reflects the emerging nature of AI research in OFP rather than a limitation of the search strategy. Establishing the current evidence base is crucial to identify methodological gaps and to guide future multicenter, prospectively validated investigations.

## 5 Conclusions

AI-based systems have demonstrated considerable potential as adjunctive tools in the diagnosis, classification, and prediction of OFP conditions. ML and DL models have shown promising performance on clinical, imaging, thermographic and neuroimaging datasets, while classical ML algorithms may often yield robust and interpretable results. However, current evidence is limited by methodological heterogeneity, retrospective single center study design, small datasets, limited external validation, and concerns regarding generalizability and explainability. Future studies should focus on prospective multicenter validation studies with standardized datasets and externally validated models. Explainable AI frameworks and collaborative research approaches can improve transparency, clinician trust, data security, and model generalizability. Systems must be prospectively validated in real-world clinical settings and evidence-based clinical decision support platforms with robust prospective clinical trials prior to regular clinical implementation.

## Data Availability

The original contributions presented in the study are included in the article/Supplementary Material, further inquiries can be directed to the corresponding author.
